# Role of Repeat Preoperative Endoscopy in Colorectal Cancer: Patients' Perspectives

**DOI:** 10.7759/cureus.108549

**Published:** 2026-05-09

**Authors:** Zacharie Cloutier, Garrett Johnson, Jessica Shanahan, Dara Hallock, Farhana Shariff, Eric Hyun, Harminder Singh, Ramzi M Helewa

**Affiliations:** 1 Department of Surgery, University of Manitoba, Winnipeg, CAN; 2 Department of Internal Medicine, University of Manitoba, Winnipeg, CAN

**Keywords:** colorectal cancer, patient perspective, preoperative plan, qualitative study, repeat endoscopy, semi-structured interview method

## Abstract

Background: Repeat preoperative endoscopy is a common practice in colorectal cancer care; however, it is often viewed as inefficient, potentially avoidable, and incurring costs or delays. While provider and system perspectives have been examined, this study aimed to explore patients' experiences and perspectives regarding repeat preoperative colonoscopy to understand its perceived value within the cancer care pathway.

Methods: We conducted a qualitative study using semi-structured telephone interviews at a tertiary academic center. Twenty adult patients treated for colorectal cancer (2019-2025) who underwent repeat preoperative endoscopy were recruited to explore their perspectives. Transcripts were analyzed using inductive thematic analysis rooted in grounded theory.

Results: Despite the procedural burden, 80% of participants reported a positive overall experience, and 75% perceived a clear benefit from the repeat procedure. Three major themes emerged: (1) Trust and Verification, where patients valued relationship-building with the surgeon and the reassuring "second look" for surgical accuracy and planning; (2) Part of the Patient Journey, where the procedure was accepted and normalized as a manageable step despite the burden of bowel preparation; and (3) Communication and Coordination, where participants highlighted gaps in care plan comprehension and logistical navigation.

Conclusions: Patients largely accepted repeat preoperative endoscopy as a justified and reassuring component of their care, fostering trust in the surgical plan, rather than perceiving it as a redundant or burdensome delay in their treatment. This counter-narrative to traditional provider-focused metrics suggests that while system efficiency remains important, repeat endoscopy provides a unique opportunity for enhancing the patient-provider therapeutic alliance.

## Introduction

Most colorectal cancers are diagnosed by colonoscopy. Prior to definitive surgery, 28.6% to 40.5% of patients undergo repeat endoscopy [1-3]. This avoidable practice carries significant consequences, including treatment delays, increased healthcare costs, patient discomfort, and exposure to procedural risks [1,3-5]. While the oncologic impact of moderate delays is debated [6-8], the associated psychological effects on patients are well recognized [9].

Repeat endoscopy is frequently driven by non-standard reporting and subsequent communication breakdown between the initial endoscopist and surgeon [10-12]. Key factors include inconsistent tattooing and documentation practices [1,3,12,13], differing provider opinions on procedural necessity [10], and issues of interprofessional trust [11]. System-level efforts to address these inconsistencies, such as standardized synoptic reporting, have yielded mixed results in reducing repeat procedures [5].

While the physician perspectives on repeat preoperative endoscopy are well documented, patient perspectives remain largely unexamined. For endoscopy in general, the burden of repeated procedures for chronic diseases is well described [14]. Prior research highlights patient priorities including managing discomfort, clear communication, and a caring patient-provider relationship [15-17]. Despite the recognition of patient-reported outcomes as a major endoscopy quality indicator [18], it remains unclear how patients perceive repeat preoperative endoscopy prior to colorectal cancer surgical resection [4]. The primary objective of this study was to address this knowledge gap through the first qualitative, interview-based exploration of patients' perspectives on repeat preoperative endoscopy prior to colorectal cancer resection.

## Materials and methods

Study design

We performed a single-institution, semi-structured interview study to explore patients' perspectives on repeat preoperative endoscopy. The study is reported according to the COREQ (Consolidated Criteria for Reporting Qualitative Research) framework [19]. Patients were contacted by telephone and provided informed consent prior to participation. Telephone-based individual interviews were conducted at the workplace exclusively by a trained non-clinician female research assistant with no prior relationship with the participants using a semi-structured interview guide created and pilot-tested by the research team (Appendix 1). Interviews were transcribed by a translation service (Translation Agency of Manitoba) certified for health information privacy and security. Field notes were taken during interviews. Transcripts confirmed the adherence to the interview guide and were not returned to participants, and no repeat interviews were conducted. The University of Manitoba Health Research Ethics Board approved the study.

Study population

Participants were recruited from St. Boniface Hospital in Winnipeg, a tertiary referral center for colorectal surgery in Manitoba, Canada. Potential participants were identified from an institutional database of adult patients who had undergone repeat preoperative endoscopy prior to surgical management of colorectal tumors. Further details on this database and study setting have been previously reported [5,20-22]. A purposive sampling strategy was used to ensure diverse perspectives representing a range of demographic and clinical variables, including tumor location, disease stage, gender, and residential setting. Of 30 patients approached, 23 consented to participate, of whom 20 successfully scheduled and completed a 30-minute interview.

Data analysis

Transcribed interviews were analyzed following the methods described by Miles et al. [23], using an inductive thematic analysis approach, a methodology rooted in grounded theory. Initially, one reviewer (ZC) assessed the transcripts to confirm that data saturation had been reached. Two independent reviewers (ZC and GJ) familiarized themselves with the data by reading all 20 transcripts repeatedly before proceeding with independent line-by-line coding to develop a preliminary codebook and identify emerging themes. The analysis then became iterative, with both reviewers meeting regularly to discuss emerging codes, compare interpretations, and regroup codes into final themes and subthemes through consensus. This process also confirmed data saturation, as codebook stability and consistency were achieved after 10-15 interviews and no new deviant cases emerged. Quantitative data were derived from a retrospective chart review and codified interview data. Software (NVivo 15, 2024, Lumivero, Denver, USA) was used to manage and organize the qualitative data.

Validity and reliability

Several recommended strategies were employed to promote the trustworthiness of the interview findings [24]. The authors and clinicians did not interact with research participants during the interviews. Peer debriefing was conducted regularly between the two primary analysts to discuss emerging themes and interpretations, with a third independent reviewer (JS) consulted to arbitrate any unresolved differences as needed. Further strategies included reflexive engagement with the data, reporting disconfirming evidence, and presenting anonymous illustrative quotes, as recommended [24].

## Results

Participant characteristics

**Figure 1:**
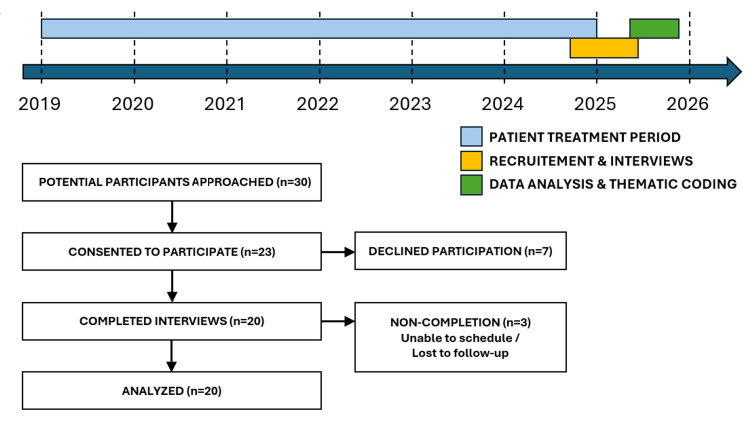
Study timeline and patient participation flowchart. Image created using MS PowerPoint.

Twenty patients (n = 9 women, 45%) participated in the study. The patient recruitment flowchart and study timeline are depicted in Figure 1. The mean participant age was 63.2 years, the majority resided in an urban setting (n=16, 80%), and most were diagnosed with rectal cancer (n=12, 60%). The cohort’s median treatment year was 2023 (2019-2025). Additional demographic and clinical characteristics are detailed in Table 1.

**Table 1:** Participant demographic and clinical characteristics (n=20).

Characteristics	Value or N (%)
Age (years)	
Mean	63.2
Range	43-82
Gender	
Male	11 (55)
Female	9 (45)
Residence	
Urban	16 (80)
Rural	4 (20)
Cancer location	
Rectal	12 (60)
Colon	8 (40)
Disease stage	
Stage 0	1 (5)
Stage I	7 (35)
Stage II	4 (20)
Stage III	8 (40)
Neoadjuvant treatment	
None	14 (70)
Chemoradiation	4 (20)
Chemotherapy	1 (5)
Total neoadjuvant therapy	1 (5)
Operation	
Low anterior resection	12 (60)
Left-sided resection	3 (15)
Right hemicolectomy	3 (15)
Total proctocolectomy	1 (5)
Abdominoperineal resection	1 (5)

Quantitative overview of patient experiences

The quantitative interpretation of patient-reported experiences with repeat endoscopy, as well as communication practices, is presented in Table 2. The overall sentiment was largely positive, with 16 patients (80%) conveying an entirely positive experience. Only two patients (10%) conveyed a neutral experience, and two (10%) had some negative perceptions. Accordingly, most patients (n=15, 75%) felt there was a clear benefit to undergoing the repeat colonoscopy. Repeat colonoscopy did not affect their opinion of their surgical care. Half of the participants reported varying degrees of communication gaps in their care pathway. While most patients (n=16, 80%) felt they were given a concrete care plan after their initial cancer diagnosis, six of these plans (37.5%) only consisted of a referral to a treating surgeon. Finally, most patients (n=15, 70%) had no issues changing doctors for the repeat endoscopy. Two patients noted the surgeon’s gender as an important factor in their care.

**Table 2:** Quantitative interpretation of patient perceptions of repeat endoscopy and communication practices.

Patients' perceptions of repeat endoscopy	N (%)
Overall sentiment	
Positive	16 (80)
Neutral	2 (10)
Negative	2 (10)
Perceived benefit	
Clear benefit	15 (75)
No benefit	1 (5)
Indifferent	4 (20)
Perception on care quality	
Did not affect opinion of care	20 (100)
Perception on changing endoscopist	
No issues	15 (75)
Welcomed change	2 (10)
Expected change	3 (15)
Perceptions on surgeon gender	
Important factor	2 (10)
Not Important	18 (90)
Endoscopy characteristics	
First endoscopy	
By Gastroenterologist	14 (70)
By Surgeon	6 (30)
For Symptoms	14 (70)
For Screening	6 (30)
Days to repeat endoscopy	
Average (days)	59.3
Median	45
Repeat endoscopy	
Flexible sigmoidoscopy	14 (70)
Colonoscopy	6 (30)
Communication	
Of cancer diagnosis	
By gastroenterologist	10 (50)
By surgeon	9 (45)
By family doctor	1 (5)
At first colonoscopy	17 (85)
Of care plan after diagnosis	
Received any plan	16 (80)
Plan was referral only	6 (30)
Of need for repeat scope	
Operating surgeon	19 (95)
Quality of communication	
No areas for improvement	10 (50)
Gaps reported	10 (50)

Qualitative overview of thematic analysis

**Figure 2:**
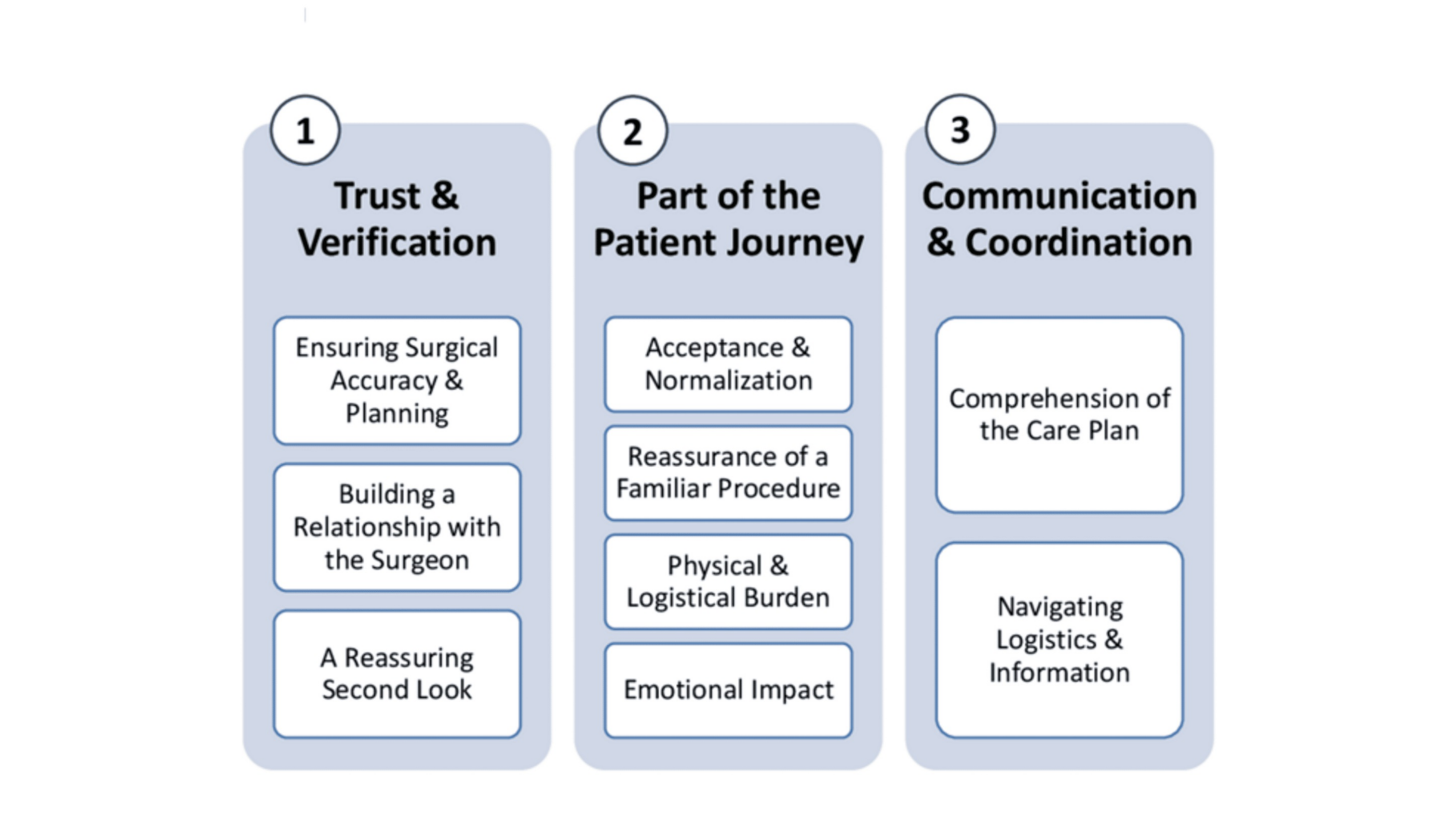
Code-based thematic hierarchy of patients' perspectives on repeat endoscopy. Image created using MS PowerPoint.

A code-based inductive thematic hierarchy based on interview data is presented in Figure 2. Three overarching themes emerged that characterized the patients' perspective on repeat preoperative endoscopy: (1) Trust and Verification, which describes how patients rationalized the repeat procedure as a necessary step for quality assurance and an opportunity to build trust with their surgeon; (2) Part of the Patient Journey, which frames the repeat endoscopy as a manageable component of a larger cancer care process; and (3) Communication and Coordination, which details the influence of logistical and informational gaps. Table 3 summarizes the patient experience.

Theme 1: Trust and Verification

The dominant theme was that patients rationalized repeat endoscopy as necessary and beneficial, which ensured surgical accuracy and built trust in their surgeon and care plan. Despite the inconvenience, repeat endoscopy was not viewed as redundant, but rather as a necessary fine-tuning tool for surgical planning and quality assurance.

Ensuring surgical accuracy and planning: Patients consistently expressed the belief that the repeat endoscopy was performed as the surgeon’s own essential preparation for the surgical procedure. This was perceived as an opportunity to “map it out” and “be prepared” by confirming the exact location and nature of the tumor. One patient captured the sentiment of accepting the burden of a repeat procedure for the benefit of surgical planning:

Yeah, colonoscopy [was] not the highlight of my day, but I still would have gone through with it again if it meant that he had a better opportunity to review and assess and to determine how he’s going to deal with it and what he was going to do.

This narrative was common, with patients referring to the procedure as a way for the surgeon to get a “better look” and to “see what they were dealing with. So it made sense”. The repeat scope was seen as a tool to “absolute[ly] pinpoint and create a plan”, providing the surgeon with a clear “game plan” before the operation. This was seen as an important step, with one patient describing it as far better than "opening up the person and kind of digging around for the spot." Overall, patients seem to take comfort that the surgeon "has taken the time to properly assess and decide the best way to move forward."

Building a relationship with the surgeon: Repeat endoscopy played a significant role in establishing a direct and strong relationship between the patient and their surgeon. Patients viewed repeat endoscopy as an opportunity for an initial hands-on interaction with the surgeon, which they found reassuring. As one patient noted, “because he would be the one in there operating on me, so I was happy with that.” This opportunity to strengthen trust was common, with one patient recalling that the surgeon wanted to “make sure I was comfortable with everything that was going on, and [that] I felt comfortable talking to him." This cherished interaction allowed surgeons to “explain the whole works” and for patients to “feel secure in what [the surgeon] was doing,” ultimately providing “peace of mind” about the surgical plan. One participant summarized this connection with the surgeon at the time of repeat endoscopy:

"Meeting me, making me feel more relaxed about going in for surgery. So, I liked it. Meeting the surgeon before walking in when I'm in a gown and says, I'll see you in the surgery in two minutes. But this felt better, seeing him before."

The repeat procedure is also a care transition that potentially involves having a provider of a different gender. While no patients reported that the gender of the second endoscopist affected their opinion of their care, one male patient reported positively on the ability to discuss sensitive issues “with a man on a man-to-man basis” while one female patient reported feelings of shame and shyness due to the male gender of the treating surgeon.

A reassuring second look: Finally, participants reported appreciation for the peace of mind provided by the opportunity to “double-check” the lesion, as a quality assurance measure. Many framed the procedure as a valuable second opinion, a confirmation of the diagnosis and the plan before proceeding with surgery. As one participant explained:

"The second colonoscopy was conducted by a colorectal specialist who specializes in these types of cases... I think it was good to have that set of eyes on it to make sure that there wasn't anything that may have been missed or anything that the first physician's expertise may not have caught."

There seems to be a shared perception that there was value for a “specialist” repeating the scope to “double confirm” the findings. One patient expressed that this direct verification "gave me that comfort level that he really knew what he was needing to do, or change his initial thought after looking for himself as to the best steps forward." Consequently, patients were often “totally in favor” of the second procedure, viewing it as a logical and necessary step to “fine tune” the plan. This second look “proved that it was the same result”, providing reassurance that “no mistakes were made” before proceeding with surgery. 

Theme 2: Part of the Patient Journey

Patients contextualized the repeat endoscopy as one of many steps within their larger, more overwhelming cancer journey. While acknowledging its burdens, particularly with repeating the bowel preparation, most viewed repeat endoscopy as an expected, manageable, and often familiar component of their overall treatment pathway.

Acceptance and normalization: For most participants, the recommendation for a repeat endoscopy was accepted with a sense of pragmatic understanding, despite the inconvenience. This blend of personal reluctance and rational acceptance was captured perfectly by one participant:

"And to do it a second time, I wasn't thrilled. But I get it."

Even while recognized as unpleasant, patients contextualized it as a necessary hurdle on the path to their definitive treatment. One patient stated: “I didn't even look at it as an inconvenience. I just looked at it as my next step.” This was a common sentiment, with others describing the procedure as just "another step in my road to wellness" and simply “something you have to go through.” This revealed a feeling of inevitability where patients “thought it was a necessary thing,” but reframed it in the context of their broader cancer treatment, with one patient explaining: “Of course, I wanted to comply with whatever was needed to have the best result, right?”

Reassurance of a familiar procedure: As all patients had previously experienced a colonoscopy, the familiarity with the process seemed to reduce the anxiety associated with the repeated procedure. The previous experience with the bowel preparation and the scope itself made the repeat endoscopy feel more manageable and less daunting. As one patient summarized:

“I really didn't have any concerns about the procedure itself, having already been through something similar. I knew what to expect.”

This prior knowledge fostered a sense of preparedness and control. Patients frequently described the second procedure as "easier because I knew what the procedures were, so there were no worries." This familiarity directly translated to reduced anxiety. One participant stated “I wasn't as nervous because now I knew what I was getting into.” Ultimately, being “more prepared for it the second time around” allowed patients to more calmly approach the repeat scope because they “knew what was happening” and felt “more relaxed.”

Physical and logistical burden: While generally accepted, patients clearly identified the logistical and physical challenges associated with a second procedure. The most frequently cited negative aspect was the need to repeat the bowel preparation, a sentiment almost universally shared among participants. One patient succinctly summarized these mixed feelings of burden and acceptance:

"[The prep], that's the worst part… I can put up with everything else."

There was a shared feeling that "the procedure itself is not all that bad. It's more so the drink.” Beyond the bowel prep, a few patients noted other logistical burdens, such as difficulty parking at a large hospital, the inconvenience of arranging travel, and taking time off work, depending on their individual circumstances. Patients described varied experiences with the changing clinical setting for the repeat scope, as some found the process “like a production line” or felt it was “a little odd being in the hallway” while others experienced a smoother process with professional staff making them “feel a little more comfortable.” Overall, these changes and challenges remained consistently framed as secondary to the primary goal of treating their cancer.

Emotional impact: The emotional response to the repeat endoscopy was overshadowed by the initial, sometimes “traumatic” shock of the cancer diagnosis for patients and their families. For most patients, the news of a repeat procedure was accepted without emotional distress, as their anxieties were focused on the cancer itself. One participant explicitly separated the procedure from its potential findings:

"I didn't really have any concerns with doing the other scope. Of course, worried about what the results might be, but I wasn't really concerned with doing another one."

While the negative emotions attached to repeating the bowel preparation were near-universal, the repeat procedure triggered specific anxieties for a minority of patients. For some, the notion of “going near the cancer site… elevated [their] anxiety and fears.” One patient worried that the repeat scope “might disrupt the cancer, might upset it somehow." This uncertainty could affect family members, with one patient’s partner thinking “it must be really bad because they are doing a second one.” Even then, these negative feelings were moderated by a resilient focus on the cancer treatment, which one participant put as “the priority in [their] life."

Theme 3: Communication and Coordination

While patients were generally accepting of repeat endoscopy, the communication surrounding both the repeat procedure and the broader care pathway was a frequent source of confusion and frustration. This theme highlights how gaps in information and coordination impacted patients' perceptions and overall experience.

Comprehension of the care plan: A key aspect of the patient journey was the evolving understanding of their overall care plan. Patients often described the initial phase after their diagnosis as a period of uncertainty, as a clear plan was not always initially provided. This left them feeling passive and unsure of what to expect. As one patient explained:

"But I wasn't really, they didn't say, okay, you're going to do this, this, this, this, and this, right. I didn't have that information, right. Just like one step at a time kind of a thing, right?"

This initial lack of clarity was commonly resolved during the consultation with the operating surgeon. Patients consistently described this meeting as an opportunity for clarification and direction. One participant recalled how the surgeon "explained everything to me," and outlined a “treatment plan that was clear.” For many, this specific consultation was the point at which they finally felt they had a roadmap for their care, with one patient stating, "I couldn’t have asked for better care at that point. But it was sort of leading up to that appointment that was a struggle."

Navigating logistics and information: In addition to understanding the overall care plan, patients also reported significant challenges in managing the volume and coordination of logistical information. Many described being "bombarded by information," receiving multiple letters, and struggling to process what was happening next, creating uncertainty and anxiety during a stressful time. This outlines the need for a clear guide for patient navigation. One participant equated the lack of such a resource to being left on their own:

"An information packet, maybe somebody calling me to explain it. That would've been very helpful. You're kind of left out on your own with your own ideas in your head about why certain things are happening and it's nice to get explanation."

Patients expressed a strong desire for more structured, tangible information to help them manage the process, such as "a printout of what's next" or a "pre-colon package," acknowledging that in the shock of receiving a cancer diagnosis, "you're not hearing it." Patients were left having to make "numerous phone calls" to navigate the logistics of scans, endoscopy, and appointments. This common experience underscores the need for improved coordination and more practical communication to help manage patient expectations and alleviate the stress of navigating the healthcare system.

**Table 3:** Dichotomy of the patient experience with repeat preoperative endoscopy.

Patient experience	Main perspective	Illustrative quote
Acceptance and willingness	Patients demonstrated a sincere assent and resilience in undergoing additional procedures, all in the pursuit of a perceived better outcome	"I'm happy. The more tests [there] were... the more information that [the surgeon] was going to have to help properly diagnose me and get me on the path to healing, I have no problem with that whatsoever. Zero."
Need for improved guidance	This acceptance was conditional, as patients strongly desired clearer communication, concrete information and a better explanation of the care process	"There really needs to be more information, more opportunity provided in terms of sort of explaining what the process is going to be like and what's happening."

## Discussion

Repeat preoperative endoscopy for colorectal cancer is common, associated with delays to definitive surgical care [2], and may be redundant in many circumstances [1,3]. Consequently, our initial hypothesis for this study was that patients would perceive repeat preoperative colonoscopy as a negative experience. Surprisingly, our results provide a notable counter-narrative. Most participants (80%) reported a positive overall experience, with many perceiving a clear benefit to repeating the scope. This unexpected finding prompted us to understand why patients often valued the repeat procedure. Three primary themes emerged that help explain this patient perspective: (1) Trust and Verification, (2) Part of the Patient Journey, and (3) Communication and Coordination. Participants predominantly viewed the repeat procedure not as a burden, but instead as a valuable opportunity and logical step that enhanced the quality and safety of their care.

A central finding of this study is the marked contrast between the provider/system perspective on repeat preoperative endoscopy and that of the patient. With rates of 29-40.5% reported, repeat endoscopy has been characterized in the literature as a problem of inefficiency, driven by gaps in interprofessional communication and nonstandard practices [1-3,11-13]. Preoperative tattooing is inconsistently applied and documented (57-58.7% of cases) [2,13], representing a critical modifiable factor for localization and a missed opportunity given its proven ability to reduce localization errors [25] (from 15.4% to 9.5%) and the odds of repeat endoscopy (OR 0.48) [2]. Providers most frequently report endoscopic tattooing or surgical planning as reasons for repeating preoperative endoscopy [1,3]. In this study, patients found the repeat endoscopy to be an opportunity to verify findings and fine-tune the surgical plan. The dominant theme of Trust and Verification aligns with the limited literature suggesting that patients may perceive repeat procedures positively as a reassuring second opinion [4]. While prior work from our group highlighted poor trust between clinicians as a driver for repeat scopes [11], our present findings indicate that, for the patient, the procedure serves to strengthen trust with their surgeon, reinforcing confidence before an operation.

A second theme emerged as patients consistently framed repeat endoscopy as a normalized Part of the Patient Journey. Although inconvenient, the procedure was an expected and manageable step relative to their overarching goal of cancer treatment. The main burden was caused by a second bowel preparation, which is consistent with other qualitative endoscopy research [14,16]. Patients accepted repeat endoscopy with resilience, driven by two subthemes: the normalization of the procedure as part of routine high-quality care, and familiarity with the procedure. While the emotional reaction to the repeat procedure was mixed, the prior experience facilitated the willingness to return, consistent with previous findings [26].

Finally, the theme of Communication and Coordination highlights clear opportunities for improvement. The findings that a portion of patients lacked comprehension of their overall care plan underscore a communication deficit consistent with existing literature emphasizing patients’ desire for clear information and the negative impact of feeling like passive recipients of care [15,17,26]. Patients repeatedly expressed the need for concrete resources to help them navigate their care pathway. The lack of a clear roadmap integrating information about repeat endoscopy and the treatment plan is a missed opportunity for shared decision-making, a collaborative process essential for ensuring that treatment aligns with patients' values and preferences [27]. Interestingly, a minority of patients highlighted the provider’s gender as a factor influencing comfort and communication, pointing to broader issues of representation and patient choice within care transitions.

Limitations

This study has several limitations. As a single-center qualitative investigation (n=20) in a public healthcare system, the findings offer a deep contextual understanding rather than statistical generalization. Experiences may differ in other healthcare systems. Our context is one characterized by high rates of repeat preoperative endoscopy, where coordinated quality improvement and research efforts between gastroenterologists and surgeons have focused on enhancing this process. Although data saturation was reached, the study is subject to recall bias due to its retrospective recruitment and potential selection bias. The predominant positive sentiment within our cohort may reflect a participation bias of patients with positive outcomes. Member checking was performed by the interviewer in real-time, with brief summaries and requests for clarification to confirm accuracy. Perceptions of patients who did not undergo repeat colonoscopy were not assessed.

## Conclusions

To our knowledge, this is the first qualitative study to explore the perspectives of colorectal cancer patients on repeat preoperative endoscopy. Our findings reveal an inherent value to the repeat scope from the patients' perspective. Participants overwhelmingly perceived the repeat procedure as a positive and beneficial step in their care pathway, despite identified burdens and communication gaps. The role of repeat preoperative endoscopy was one of quality assurance and trust-building, met with acceptance and resilience. Therefore, while reducing unnecessary procedures remains a worthy objective in a resource-limited setting, repeat preoperative endoscopy may in fact strengthen the therapeutic alliance and improve shared decision-making.

## Appendices

Appendix 1: Interview Script

Study Title: Role of Repeat Preoperative Endoscopy in Colorectal Cancer: Patients' Perspectives

Objectives  

⦁    Attain a better understanding of patients' opinions around having multiple endoscopy procedures.

⦁    Explore the potentially negative and positive experiences with repeat endoscopy.   

⦁    Identify areas for possible improvements in patient-centered care, specifically in patients who have colorectal cancer, undergoing multiple endoscopy procedures.

Interview Script

Introduction

My name is __________ and I am a _______________. Thank you for volunteering for this study. We want to hear about your experiences with colonoscopies before surgery. Specifically, we want to ask questions regarding undergoing repeat endoscopy procedures in patients who have had elective surgery for their colorectal cancer in Winnipeg, Manitoba. Our goal is to make care better for people with colorectal cancer by improving communication between patients and specialists and finding ways to make colonoscopies before surgery better. Please share your thoughts and answer questions as you like. There are no right or wrong answers. We are gathering ideas and thoughts about colonoscopies before surgery to see if we can better understand the patient experience.

To assist in data collection of the ideas and thoughts, we will be recording this conversation. Any recorded files will be stored on a password-protected computer and will be accessed by a PHIA compliant transcription service. Data will be accessed only by the research team and will be securely stored for up to five years.

Thank you for participating!

We will begin recording now.

Focusing Statement

Today, we want to talk about your experience with colorectal cancer, especially your colonoscopy procedures before the cancer surgery. In Canada, colorectal cancer is the second most common cancer. A colonoscopy is the main test to check for and diagnose colon tumors. We want to hear from patients like you about what it is like to have multiple colonoscopies before surgery. Your opinions will help us figure out how to make things better for people with colorectal cancer. Having colorectal cancer can be a difficult experience. Our goal for this study is to improve how we care for colorectal cancer patients who have repeated colonoscopies.

Questions:

1.     How was your colon or rectal cancer first discovered?

a.    What kind of symptoms were you having?

b.    What investigations were recommended?

c.    What was your understanding of the reason for your initial colonoscopy before cancer surgery?

2.    What can you tell me about your experience with the initial colonoscopy during your cancer diagnosis?

a.    Probes:

       (i)  How long did you wait for your endoscopy date?

       (ii)  How far from your home was your first colonoscopy?

       (iii) Did you experience pain?

       (iv) How inconvenient was the colonoscopy for you?

       (v)  Did you have any issues, concerns or complications during the procedure?

       (vi) Did you have any issues after the procedure?

One of the goals of this research is to understand and improve the way people are informed about their cancer diagnosis and why they need multiple colonoscopies

3.    Who informed you of your cancer diagnosis (endoscopist, family doctor, someone else)?

a.    Did they have a plan that was laid out for you?

b.    If so, what was your understanding of that plan?

4.    What was your experience with learning about your cancer diagnosis?

5.    Who told you that you would be having another colonoscopy before surgery?

a.    What was the reason for doing another colonoscopy?

6.    What information did you receive at the time about your cancer diagnosis and the need for another colonoscopy?

a.    Did you feel you got enough information?

7.     What could have improved how the information about your cancer diagnosis and the need for another colonoscopy was shared with you?

8.    What were your concerns at that time related to the diagnosis and doing a second colonoscopy?

9.    What were your thoughts about doing another colonoscopy?

a.    (Probe issues of trust and confidence) Why do you think the surgeon/endoscopist wanted to repeat the colonoscopy?

b.    Do you think that there were additional reasons not shared with you that the surgeon/endoscopist wanted to repeat the colonoscopy?

c.    Did the plan for a repeat colonoscopy affect your opinions about your surgical care?

10.  What can you tell me about your experience with the repeat colonoscopy during your cancer diagnosis?

a.    Probes:

       (i)   How long did you wait for your endoscopy date?

       (ii)  How far from your home was your first colonoscopy?

       (iii) Did you experience pain?

       (iv) How inconvenient was the colonoscopy for you?

       (v)  Did you have any issues, concerns or complications during the procedure?

       (vi) Did you have any issues after the procedure?

11.  How did the repeat colonoscopy compare with the first one?

12.  For your repeat colonoscopy, was it with a different physician?

a.    If so, how did you feel about this?

13.  Reflecting back, what do you feel was the benefit, if any, from repeating the colonoscopy?

14.  What impact, if any, do you feel your gender had on your experience with the colonoscopies, the communication you received and your overall care?

15.  What changes would have improved the experience of the colonoscopies or communication you received in your overall care?

16.  If there was one thing you could tell the team who was involved in doing these colonoscopies, what would it be?

17.  Is there anything you would like to add?

## References

[1] Al Abbasi T, Saleh F, Jackson TD, Okrainec A, Quereshy FA (2014). Preoperative re-endoscopy in colorectal cancer patients: an institutional experience and analysis of influencing factors. Surg Endosc.

[2] Hershorn O, Park J, Singh H, Clouston K, Vergis A, Helewa RM (2021). Predictors and rates of prior endoscopic tattoo localization amongst individuals undergoing elective colorectal resections for benign and malignant lesions. Surg Endosc.

[3] Chen MZ, Devan Nair H, Saboo A (2022). A single centre audit: repeat pre-operative colonoscopy. ANZ J Surg.

[4] Choi WJ, Cleghorn MC, Quereshy FA (2016). Preoperative repeat endoscopy for colorectal cancer: what is its role and when is it necessary?. Can J Surg.

[5] Johnson GGRJ, Singh H, Vergis A, Park J, Hershorn O, Hochman D, Helewa RM (2022). Repeat preoperative endoscopy after regional implementation of electronic synoptic endoscopy reporting: a retrospective comparative study. Surg Endosc.

[6] Bondzi-Simpson A, Sutradhar R, Wright FC (2025). Association of traditional and new surgical wait time targets and survival for curative-intent colorectal cancer surgery: a population-based cohort study. Ann Surg.

[7] Simunovic M, Rempel E, Thériault M-E, Baxter NN, Virnig BA, Meropol NJ, Levine MN (2009). Influence of delays to nonemergent colon cancer surgery on operative mortality, disease-specific survival and overall survival. Can J Surg.

[8] Grass F, Behm KT, Duchalais E (2020). Impact of delay to surgery on survival in stage I-III colon cancer. Eur J Surg Oncol.

[9] Derry-Vick HM, Heathcote LC, Glesby N, Stribling J, Luebke M, Epstein AS, Prigerson HG (2023). Scanxiety among adults with cancer: a scoping review to guide research and interventions. Cancers (Basel).

[10] Azin A, Jimenez MC, Cleghorn MC, Jackson TD, Okrainec A, Rossos PG, Quereshy FA (2016). Discrepancy between gastroenterologists' and general surgeons' perspectives on repeat endoscopy in colorectal cancer. Can J Surg.

[11] Hershorn O, Park J, Singh H, Restall GJ, Clouston KM, Vergis AS, Helewa RM (2023). Variability in communication and reporting practices between gastroenterologists and general surgeons contributes to repeat preoperative endoscopy for colorectal neoplasms: a qualitative analysis. Dis Colon Rectum.

[12] Johnson G, Singh H, Helewa RM, Sibley KM, Reynolds KA, El-Kefraoui C, Doupe MB (2024). Gastroenterologist and surgeon perceptions of recommendations for optimal endoscopic localization of colorectal neoplasms. Sci Rep.

[13] Nahid M, Shrestha AK, Imtiaz MR, Basnyat PS (2020). Endoscopic tattooing for colorectal lesions: impact on quality of care and patient outcomes. Ann R Coll Surg Engl.

[14] Ryhlander J, Ringstrom G, Simrén M, Stotzer P-O, Jakobsson S (2019). Undergoing repeated colonoscopies - experiences from patients with inflammatory bowel disease. Scand J Gastroenterol.

[15] Dubois H, Creutzfeldt J, Törnqvist M, Bergenmar M (2020). Patient participation in gastrointestinal endoscopy - from patients' perspectives. Health Expect.

[16] Shamim S, Andresen YLM, Vind Thaysen H, Hovdenak Jakobsen I, Nielsen J, Kjaergaard Danielsen A, Konradsen H (2021). Experiences of patients undergoing bowel preparation and colonoscopy: a qualitative longitudinal study. J Multidiscip Healthc.

[17] Restall G, Michaud V, Walker JR (2020). Patient experiences with colonoscopy: a qualitative study. J Can Assoc Gastroenterol.

[18] Rosvall A, Annersten Gershater M, Kumlien C, Toth E, Axelsson M (2022). Patient-reported experience measures for colonoscopy: a systematic review and meta-ethnography. Diagnostics (Basel).

[19] (2025). Consolidated Criteria for Reporting Qualitative Research (COREQ): a 32-item checklist for interviews and focus groups | EQUATOR Network. https://www.equator-network.org/reporting-guidelines/coreq/.

[20] Roy H, Johnson G, Singh H, Hyun E, Moffatt D, Vergis A, Helewa R (2024). Implementation of synoptic reporting for endoscopic localization of complex colorectal neoplasms. Cureus.

[21] El-Kefraoui C, Johnson G, Singh H, Helewa RM (2023). Optimal endoscopic localization of colorectal neoplasms: a comparison of rural versus urban documentation practices. World J Surg Oncol.

[22] Johnson GG, Hershorn O, Singh H, Park J, Helewa RM (2022). Sampling error in the diagnosis of colorectal cancer is associated with delay to surgery: a retrospective cohort study. Surg Endosc.

[23] Miles MB, Huberman AM, Saldana J (2014). Qualitative Data Analysis: A Methods Sourcebook, Third Edition. https://www.metodos.work/wp-content/uploads/2024/01/Qualitative-Data-Analysis.pdf.

[24] Ravitch SM, Carl NM (2019). Qualitative Research: Bridging the Conceptual, Theoretical, and Methodological.

[25] Acuna SA, Elmi M, Shah PS, Coburn NG, Quereshy FA (2017). Preoperative localization of colorectal cancer: a systematic review and meta-analysis. Surg Endosc.

[26] Loftus R, Nugent Z, Graff LA, Schumacher F, Bernstein CN, Singh H (2013). Patient satisfaction with the endoscopy experience and willingness to return in a central Canadian health region. Can J Gastroenterol.

[27] Abelson JS, Gaetani RS, Hawkins AT (2025). Shared decision making in the treatment of rectal cancer. J Clin Med.

